# Preoperative dual-energy computed tomography and positron-emission tomography evaluation of lymph node metastasis in esophageal squamous cell carcinoma

**DOI:** 10.1371/journal.pone.0309653

**Published:** 2024-09-20

**Authors:** Xuyang Sun, Tetsu Niwa, Toshiki Kazama, Takashi Okazaki, Kazuo Koyanagi, Nobue Kumaki, Jun Hashimoto, Soji Ozawa

**Affiliations:** 1 Department of Diagnostic Radiology, Tokai University School of Medicine, Isehara, Japan; 2 Department of Gastroenterological Surgery, Tokai University School of Medicine, Isehara, Japan; 3 Department of Pathology, Tokai University School of Medicine, Isehara, Japan; 4 Department of Surgery, Tamakyuryo Hospital, Machida, Japan; National Institute of Cancer Research, TAIWAN

## Abstract

**Purpose:**

To investigate the detectability of lymph node metastasis in patients with esophageal squamous cell carcinoma using a combination of dual-energy computed tomography (CT) and positron-emission tomography (PET) parameters.

**Methods:**

We analyzed dual-energy CT and PET preoperative data in 27 consecutive patients with esophageal squamous cell carcinoma (23 men, 4 women; mean age, 73.7 years). We selected lymph nodes with a short-axis diameter of ≥5 mm and measured CT values, iodine concentrations, fat fractions, long- and short-axis diameters, and ratio of long- and short-axis diameters. We performed visual assessment of lymph node characteristics based on dual-energy CT and determined the maximum standardized uptake value via PET. The measured values were postoperatively compared between pathologically confirmed metastatic and nonmetastatic lymph nodes. Stepwise logistic regression analysis was performed to determine factors associated with lymph node metastasis. Diagnostic accuracy was assessed via receiver operating characteristic curve analysis.

**Results:**

Overall, 18 metastatic and 37 nonmetastatic lymph nodes were detected. CT values, iodine concentrations, fat fractions, and the maximum standardized uptake values differed significantly between metastatic and nonmetastatic lymph nodes (*p* < 0.05). Stepwise logistic regression showed that iodine concentration and the maximum standardized uptake value were significant predictors of metastatic lymph nodes. The areas under the curve of iodine concentrations and maximum standardized uptake values were 0.809 and 0.833, respectively. The area under the curve of the combined parameters was 0.884, with 83.3% sensitivity and 86.5% specificity.

**Conclusion:**

Combined dual-energy CT and PET parameters improved the diagnosis of lymph node metastasis in patients with esophageal cancer.

## Introduction

Esophageal cancer, which is aggressive and malignant, is the sixth leading cause of cancer death and the eighth most common cancer globally [1, 2], with an increasing incidence, poor prognosis, and 5-year survival rate of 15%–25% [1–3]. It is histologically divided into two main types: squamous cell carcinoma and adenocarcinoma [4]. The predominant histological subtype is adenocarcinoma in Europe and the United States and squamous cell carcinoma in parts of Asia [1]. Lymph node (LN) metastases occur more frequently in esophageal cancer than in other gastrointestinal cancers [5, 6]. LN metastases of esophageal cancer are primarily distributed in three regions: neck, mediastinal, and abdominal regions. The LN metastatic status is a key factor affecting the postoperative outcome in patients with esophageal cancer. In particular, the number of LN metastases is an important risk factor for poor outcomes because the prognosis of esophageal cancer is worse in patients with multiple LN metastases than in those with a single LN metastasis [5]. Early identification of LN metastasis is crucial in determining treatment strategies [7]. Several imaging tools, such as computed tomography (CT) and endoscopic ultrasonography, are frequently used to detect LN metastasis in clinical practice. Although these imaging techniques can be used to assess the disease extent, the detection of LN metastases has several limitations [8–10].

CT is currently the most common method for examining LN metastasis of esophageal cancer. However, the detection of LN metastasis of esophageal cancer via conventional CT is sometimes challenging [11, 12]. Dual-energy CT uses data from two different X-ray spectra, typically low- (80 kVp) and high- (140–150 kVp) energy spectra [13]. Considering that different materials have distinct characteristics, dual-energy CT can provide mixed-energy, iodine, virtual noncontrast, and virtual monoenergertic images [14, 15]. The utility of dual-energy CT for evaluating LN metastasis has been reported in various cancers, such as colorectal, gynecological, hypopharyngeal, lung, and liver cancers [14, 16–20]. Recently, ^18^F-fluorodeoxyglucose (FDG) positron-emission tomography (PET) has commonly been used for diagnosis, initial staging, restaging, prognostication, and treatment monitoring in various cancers [10, 21, 22]. However, the role of FDG PET in detecting LN metastasis of esophageal cancer remains controversial [10, 23]. The ability to detect functional abnormalities of glucose metabolism is a key factor that distinguishes FDG PET from CT. This is an important factor because when it is challenging to make judgments based on the shape of an organ, the diagnostic accuracy can be improved by assessing the organ’s function. However, PET has a poor inherent spatial resolution of approximately 5 mm [11]. Therefore, we hypothesized that combined dual-CT and PET parameters can improve the diagnosis of LN metastasis of esophageal cancer.

This study aimed to assess the ability of combined dual-energy CT and PET parameters to detect preoperative LN metastases in patients with esophageal squamous cell carcinoma.

## Materials and methods

### Patients

This retrospective study was approved by the Institutional Review Board for Clinical Research, Tokai University, which also waived the need for informed consent (IRB No. 21R292). This study searched for patients with esophageal cancer who were assessed via the radiologic reporting system. The selected patients were initially examined via dual-energy CT and PET. The inclusion criteria were as follows: 1) patients who underwent esophagectomy without any preoperative treatment from March 2017 to February 2022 and 2) those with pathologically confirmed squamous cell carcinoma. Dual-energy CT and PET images were reviewed on an in-house workstation. The observers only assessed the dual-energy CT or PET images and were blinded to other images or medical records. The measured data were retrieved for analysis on December 20, 2022, without any identifiable patient information.

### CT

CT was performed using a dual-energy scanner (SOMATOM Force, Siemens Healthcare, Forchheim, Germany). For contrast-enhanced CT, the contrast agent was administered via an injector at a dose of 23.0 mg I/body weight (kg)/s for 20 s. The amount of contrast agent was determined based on the patient’s body weight (<57 kg, 300 mg I/mL; 57–66 kg, 320 mg I/mL; and >66 kg, 370 mg I/mL). Dual-phase contrast-enhanced CT images were obtained; the early-phase images were acquired at the determined time via the bolus-tracking method, whereas the late-phase images were acquired 2 min after the injection of contrast agent. Early-phase images ranged from the top of the chest to diaphragm, whereas late-phase images ranged from the neck to pelvis. The following dual-energy CT scan parameters were employed: tube voltage, 90 and 150 kVp; gantry rotation time, 0.25 s; pitch, 0.55; slice collimation, 192 × 0.6 mm; and automatic current modulation (CARE Dose 4D). Before CT, the patient was asked to hold their breath. Electrocardiogram (ECG) gating was not used. Scan data reconstruction was performed using a Bf40 soft-tissue convolution kernel and iterative reconstruction (ADMIRE strength level 1) based on a 350-mm field of view (FOV) and 512 × 512 matrix, with 1-mm slice thickness and 0.8-mm increment.

### PET

TruePoint Biograph16 (Siemens Healthineers, Erlangen, Germany) was used to perform PET/CT. Before receiving FDG injection, the patients were instructed to fast for ≥5 h. Subsequently, images were obtained from the skull base to mid-thigh 50 min post-intravenous injection of 3.7 MBq/kg FDG. Initially, 16-slice CT (130 kV, quality reference mAs, 60) with 5-mm slice thickness was performed for attenuation correction and anatomy localization. After CT, 3D PET was performed for 2 min/field with an axial FOV of 70 cm. An iterative algorithm with 3 iterations, 21 subsets, and 168 × 168 matrix was used to reconstruct the PET images.

### Imaging analysis

Late-phase CT images were transferred to a workstation (*syngo*.*via*, 8.04, Siemens Healthcare, Forchheim, Germany). Initially, LNs with a short-axis diameter of ≥5 mm were determined by an experienced radiologist (TN, 25 years of experience in chest imaging). These LNs were analyzed via postoperative CT and confirmed as “resected” or “nonresected.” Thus, preoperatively identifiable LNs with short-axis diameter of ≥5 mm that were postoperatively confirmed as “nonresected” were excluded.

To analyze CT images, an iodine map was constructed on the workstation, and a circular region of interest was manually drawn, as large as possible, within each LN by the radiologist (TN). An iodine subtraction algorithm (Liver VNC, Siemens Healthcare) was used to calculate the iodine concentration (IC) and fat fraction. The CT values were determined by mixing 60% and 40% of the low-and high-keV CT values. The long- and short-axis diameters of each LN were also measured on the workstation. The second-time measurements were performed by the same radiologist after >3 weeks. The final parameters were determined by averaging these two measurements. To assess interobserver agreements, another radiologist (TO, 12 years of experience in chest imaging) assessed the CT images for 10 patients. In addition, LN characteristics, such as the presence or absence of necrosis, calcification, unclear margins, and lobulated shape in each LN, were visually assessed by two radiologists (TN and TO); these characteristics were determined via consensus between two observers.

For PET analysis, the reconstructed attenuation-corrected PET images, CT images, and fused images of matching PET and CT were available for review in axial, coronal, and sagittal planes with maximum-intensity projections. The maximum standard uptake (SUVmax) values of LNs were measured by drawing a volume of interest (VOI) on axial-plane PET images for semiquantitative analysis by an experienced radiologist (TK, 19 years of experience in PET interpretation). VOI was placed over the area of maximum activity within LNs, and SUVmax was determined as the highest SUV of the pixels within a VOI.

### Statistical analysis

LN metastasis was detected via postoperative pathological examination. We matched LNs on images, and pathological diagnosis based on LN numbers was performed by the surgeons according to the Japanese classification of esophageal cancer, 11^th^ edition [24, 25]. Imaging parameters including CT value, IC, fat fraction, long- and short-axis diameters, and ratio of long-axis to short-axis diameters as well as imaging characteristics including necrosis, calcification, unclear margins, lobulated shape, and SUVmax were compared between pathologically confirmed metastatic and nonmetastatic LNs. In addition, these parameters were compared according to the location of LNs, such as the neck, supraclavicular area, mediastinum, hilum, axilla, and intra-abdominal region. Normally distributed data were assessed via independent sample *t*-test, and nonnormally distributed data were assessed via the Mann–Whitney test. Categorical data were assessed based on the Fisher’s exact test. Interobserver agreement was evaluated using intraclass correlation coefficients (ICCs) according to the following criteria: <0.5, poor reliability; 0.5–0.75, moderate reliability; 0.75–0.9, good reliability; and >0.9, excellent reliability [26]. The correlation between IC and SUVmax in metastatic and nonmetastatic LNs was evaluated via Spearman correlation.

To determine independent predictors of LN metastasis among these parameters, stepwise logistic regression analysis was performed. For the determined predictors, receiver operating characteristic (ROC) analysis was used to assess the diagnostic accuracy. The combination of these parameters was also analyzed. The cutoff values, sensitivity, specificity determined according to the Youden Index, and area under the ROC curve (AUC) were calculated.

MedCalc Statistical Software version 22.006 (MedCalc Software Ltd., Ostend, Belgium; https://www.medcalc.org) was used to perform all statistical analyses. *p*-values of <0.05 were considered to indicate statistical significance. Normally distributed data were presented as mean ± standard deviation, whereas nonnormally distributed data were presented as median (interquartile range).

## Results

**Fig 1:**
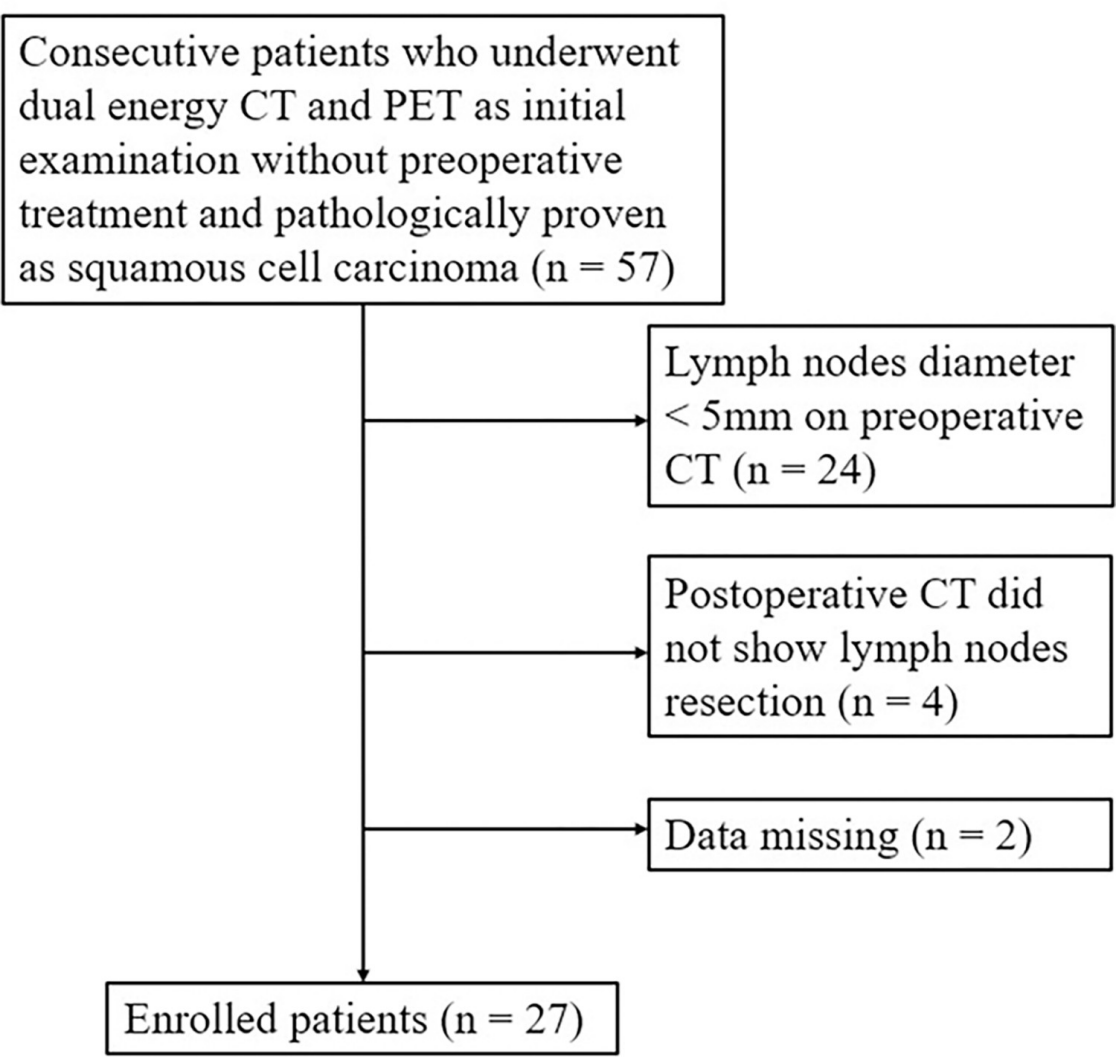
Patient-selection flowchart. Twenty-seven patients with squamous cell esophageal carcinoma were enrolled. CT: computed tomography; PET: positron-emission tomography.

Initially, 57 consecutive patients who underwent esophagectomy without presurgical treatment and had pathologically confirmed squamous cell carcinoma were included in this study. A total of 24, 4, and 2 patients were excluded because of the following respective reasons: CT did not show LNs with a short-axis diameter of ≥5 mm, postoperative CT did not reveal resection of the targeted LNs, and dual-energy CT data were missing. Finally, 27 patients (23 men, 4 women; mean age, 73.7 [range: 56–84] years) were enrolled in this study (Fig 1). Dual-energy CT data of 5 of 27 patients were derived from a previous study [27], and data regarding the other 22 patients were newly analyzed. All PET data were newly assessed.

Patients’ tumor stages were assessed according to the 8^th^ edition of the Union for International Cancer Control TNM staging [28]. Patients’ clinical characteristics and tumor stages are summarized in Table 1. Overall, 55 LNs from the 27 included patients were analyzed. In total, 18 metastatic and 37 nonmetastatic LNs were detected. The mean number of analyzed LNs for each patient was 2 (range, 1–5).

**Table 1:** Patients’ characteristics.

Characteristics	Value*
Patient	27
Age (mean [range] years)	73.7 (56–84)
Sex	
Male	23
Female	4
Primary tumor location	
Upper thoracic	5
Middle thoracic	12
Lower thoracic	10
Clinical stage	
Ⅰ	11
Ⅱ	1
Ⅲ	15
Pathological stage	
Ⅰ	7
Ⅱ	9
Ⅲ	10
Ⅳ	1

*Number of patients, otherwise indicated

**Fig 2:**
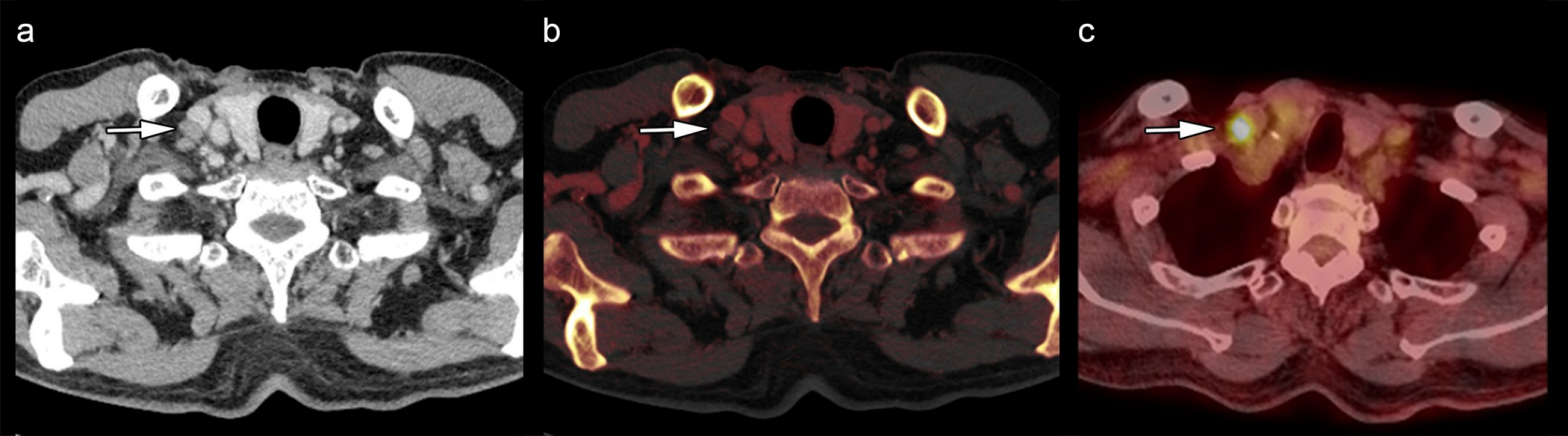
Representative dual-energy CT and PET images in patients with lymph node metastasis. Contrast-enhanced CT image (a), iodine map (b), and PET image (c) of an 83-year-old man with esophageal cancer; images show swelling of the right supraclavicular lymph node (a, b, c arrows), which was confirmed as metastasis postoperatively. The low iodine concentration (1.45 mg/mL) and high maximal standardized uptake value (10.7) were measured. CT: computed tomography; PET: positron-emission tomography.

**Fig 3:**
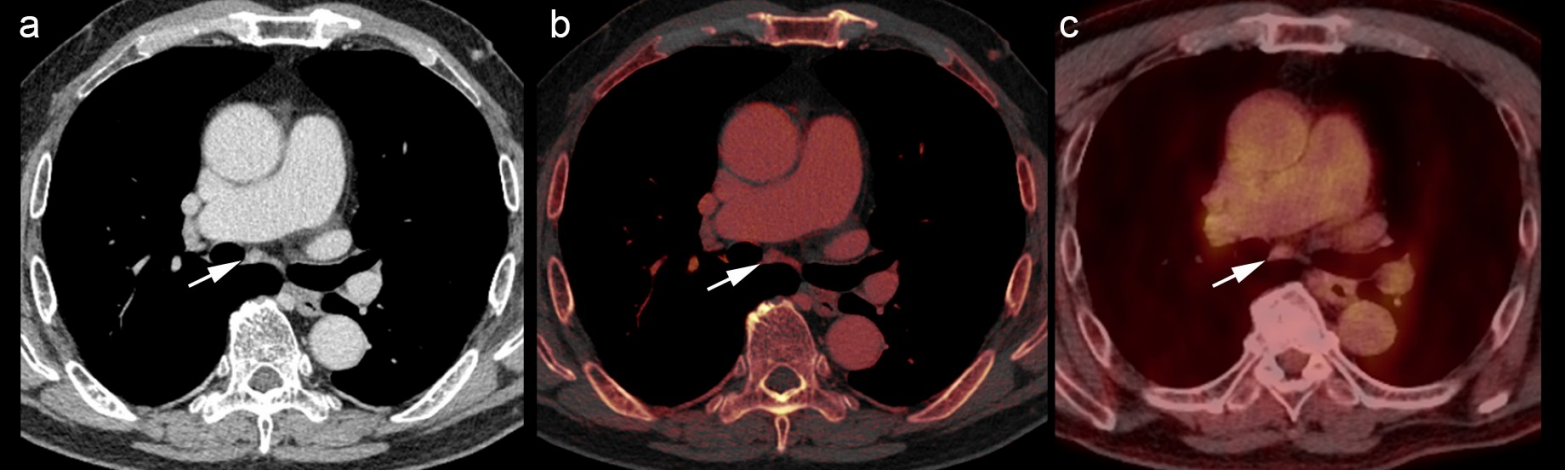
Representative dual-energy CT and PET images in patients without lymph node metastasis. Contrast-enhanced CT image (a), iodine map (b), and PET image (c) of a 76-year-old man with esophageal cancer; images show the presence of subcarinal lymph node (a, b, c arrows), which was demonstrated as nonmetastatic postoperatively. The high iodine concentration (3.35 mg/mL) and low maximal standardized uptake value (1.4) were measured. CT: computed tomography; PET: positron-emission tomography.

**Fig 4:**
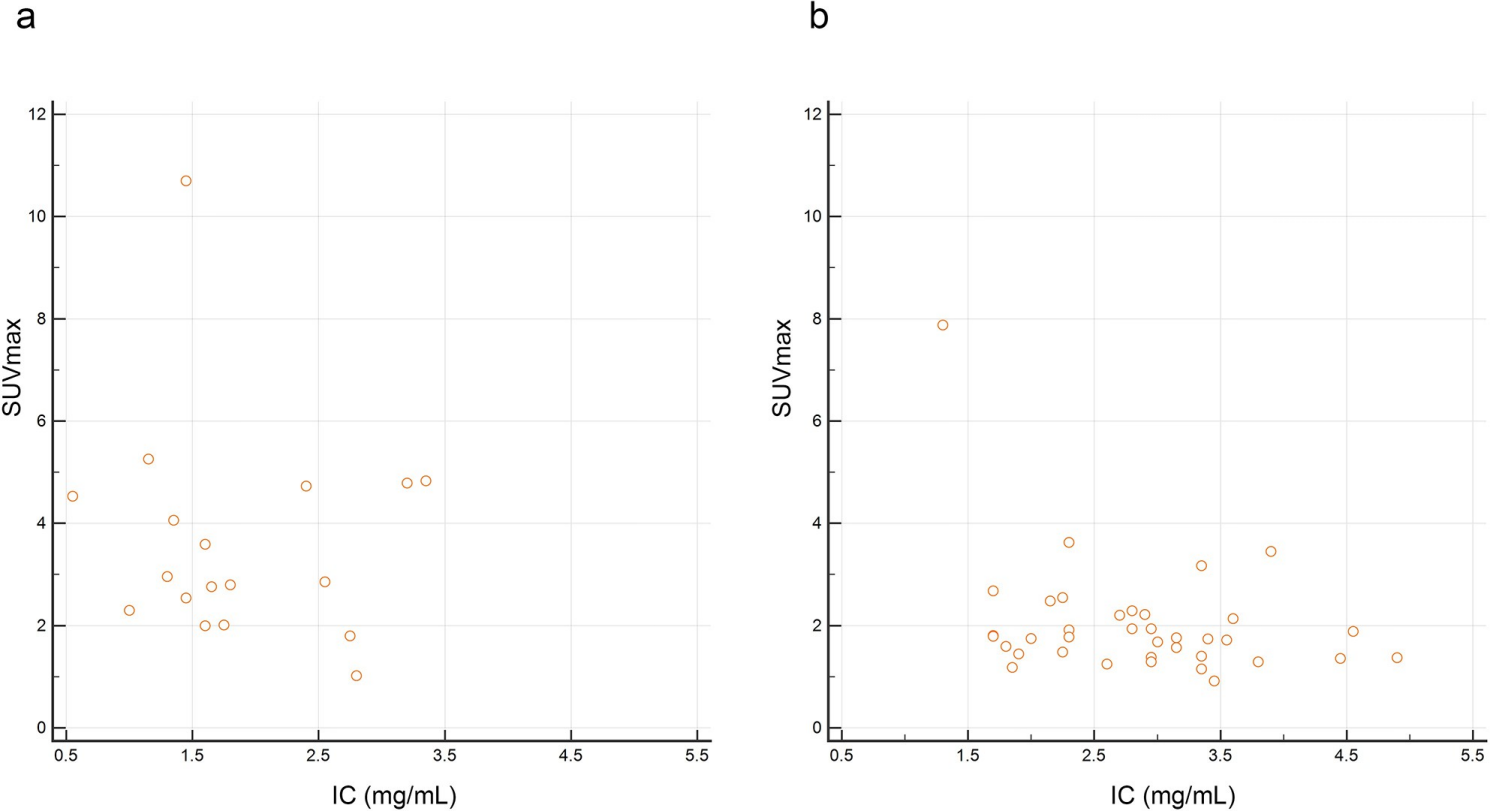
Scatterplots illustrate the correlation between iodine concentration (IC) and maximal standardized uptake value (SUVmax) in metastatic (a, Spearman ρ = −0.142, p = 0.575) and nonmetastatic (b, Spearman ρ = −0.270, p = 0.106) lymph nodes.

The mean (range) values of parameters including CT value, IC, fat fraction, long-axis diameter, short-axis diameter, ratio of long-axis to short-axis diameters, and SUVmax were 80.79 (47.7–112.8) HU, 2.52 (0.55–4.90) mg/mL, 23.4% (3.55%–51.0%), 10.2 (4.8–20.7) mm, 6.0 (4.2–10.9) mm, 1.70 (1.0–3.3), and 2.55 (0.92–10.7), respectively. CT value, IC, and fat fraction were normally distributed, whereas the long- and short-axis diameters, ratio of long-axis to short-axis diameters, and SUVmax were nonnormally distributed. Table 2 shows the comparison of dual-energy CT and PET parameters between metastatic and nonmetastatic LNs. CT values of metastatic LNs were significantly lower than those of nonmetastatic LNs (73.1 ± 15.3 vs. 84.5 ± 12.7 HU, *p* = 0.0051). ICs of metastatic LNs were significantly lower than those of nonmetastatic LNs (1.87 ± 0.78 vs. 2.84 ± 0.86 mg/mL, *p* = 0.0002). The fat fractions of metastatic LNs were significantly lower than those of nonmetastatic LNs (18.1 ± 8.6 vs. 26.0 ± 9.4%, *p* = 0.0038). The long- and short-axis diameters of metastatic LNs tended to be large; however, there was no significant difference between metastatic and nonmetastatic LNs. The ratio of long- and short-axis diameters did not differ significantly between metastatic and nonmetastatic LNs. There were no significant association between visual imaging findings and pathological findings. The SUVmax values of metastatic LNs were significantly higher than those of nonmetastatic LNs (2.91 [2.40–4.73] vs. 1.76 [1.40–2.21], *p* = 0.0001). The location of lymph nodes included neck (n = 1), supraclavicular area (n = 5), mediastinum (n = 24), hilum (n = 2), axilla (n = 0), and intra-abdominal region (n = 23). S1 Table shows the comparison based on the LN location. Figs 2 and 3 illustrate representative cases. ICCs based on CT parameters ranged from 0.9433 to 0.9711, indicating excellent reliability. No significant correlation was noted between IC and SUVmax in both metastatic (Spearman ρ = −0.142, *p* = 0.575) and nonmetastatic (Spearman ρ = −0.270, *p* = 0.106) LNs (Fig 4).

**Table 2:** Comparison of dual-energy CT and PET parameters between metastatic and nonmetastatic lymph nodes.

Parameters	Metastatic LNs (n = 18)	Nonmetastatic LNs (n = 37)	*p*
CT value (HU)	73.1 ± 15.3	84.5 ± 12.7	0.0051
IC (mg/mL)	1.87 ± 0.78	2.84 ± 0.86	0.0002
Fat fraction (%)	18.1 ± 8.6	26.0 ± 9.4	0.0038
Long-axis diameter (mm)	11.2 (7.1–13.2)	8.9 (7.3–12.0)	0.3555
Short-axis diameter (mm)	6.6 (5.1–8.0)	5.3 (4.8–6.1)	0.0818
Ratio of long-axis to short-axis diameters	1.59 (1.32–1.83)	1.68 (1.34–1.93)	0.4955
Necrosis*			0.0975
Present	3	1	
Absent	15	36	
Calcification*			–
Present	–	–	
Absent	18	37	
Unclear margin*			0.0975
Present	3	1	
Absent	15	36	
Lobulated shape*			0.3165
Present	3	2	
Absent	15	35	
SUVmax	2.91 (2.30–4.73)	1.76 (1.40–2.21)	0.0001

CT, computed tomography; PET, positron-emission tomography; IC, iodine concentration; SUVmax, maximum standardized uptake value

*Number of lymph nodes

CT value, IC, and fat fraction data with normal distributions are presented as the mean ± standard deviation. The short-axis diameter and SUVmax with nonnormal distributions are presented as the median value (interquartile range). There were significant differences in terms of CT value, IC, fat fraction, and SUVmax and no significant difference in terms of short-axis diameters between metastatic and nonmetastatic LNs.

We performed a stepwise logistic regression analysis using the independent variables that showed significant differences between metastatic and nonmetastatic LNs: CT value, IC, fat fraction, and SUVmax. This analysis revealed that IC (odds ratio [OR], 0.29) and SUVmax (OR, 1.93) were independent predictors of LN metastasis.

**Fig 5:**
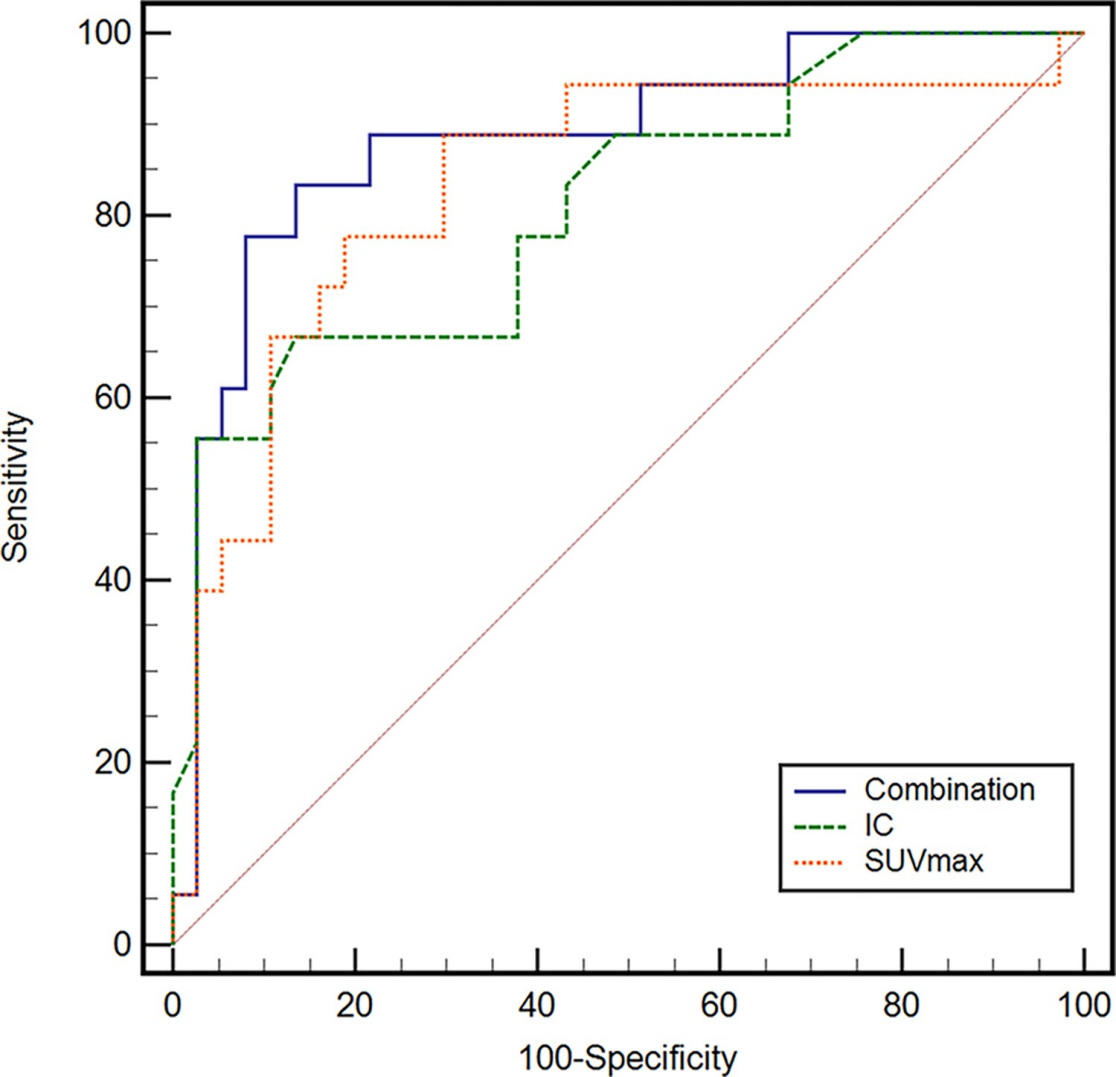
Determination of the diagnostic ability of receiver operating characteristic (ROC) curves using iodine concentration (IC), maximal standardized uptake value (SUVmax), and combination of these factors to predict lymph node metastasis of esophageal cancer. The areas under the ROC curve (95% confidence interval) of IC, SUVmax, and combination of these factors were 0.809 (0.680–0.902), 0.833 (0.708–0.920), and 0.884 (0.769–0.955), respectively.

Furthermore, ROC analysis was performed for assessing IC, SUVmax, and combination of these factors (Fig 5). The cutoff value, sensitivity (95% confidence interval [CI]), and specificity (95% CI) of IC were 1.8 mg/mL, 66.7% (41.0%–86.7%), and 86.5% (71.2%–95.5%), and those of SUVmax were 1.94, 88.9% (65.3%–98.6%), and 70.3% (53.0%–84.1%), respectively. AUCs (95% CI) of IC and SUVmax were 0.809 (0.680–0.902) and 0.833 (0.708–0.920); none of them differed significantly between metastatic and nonmetastatic LNs (*p* = 0.763). The combined IC + SUVmax model showed the best performance, with an AUC, sensitivity, and specificity of 0.884 (0.769–0.955), 83.3% (58.6%–96.4%), and 86.5% (71.2%–95.5%), respectively, which were significantly higher than those of IC (*p* = 0.0300) and nonsignificantly higher than those of SUVmax (*p* = 0.380).

## Discussion

Based on dual-energy CT, this study showed that the CT value, IC, and fat fraction of metastatic LNs were significantly lower than those of nonmetastatic LNs. The lower CT value and IC for metastatic LNs may be related to the reduced blood flow in LNs. An iodine map based on dual-energy CT can be constructed to evaluate the tissue iodine content. In this study, lower IC was detected in metastatic LNs, potentially due to vascular insufficiency or reduced blood flow in metastatic LNs. The present study results are in agreement with those of previous reports, showing lower IC in metastatic cervical LNs of squamous cell carcinoma [14]. Other reports have also shown lower IC in metastatic LNs of rectal cancer [29] as well as lung and gynecological malignancies [16]. In this study, the fat fraction of metastatic LNs was significantly lower than those of nonmetastatic LNs. This finding may be explained by the reduction in the size of normal fatty hilum in the metastatic LNs infiltrated by tumor cells [30], resulting in a low fat percentage on dual-energy CT images [31]. Consistent with our previous findings [27], the fat fraction can be used as a predictor of the presence of metastasis in LNs. In the current study, there was no significant difference in the long- and short-axis diameters, and the ratio of long-axis to short-axis diameters between metastatic and nonmetastatic LNs. Although large LNs are typically believed to have undergone metastasis, the size of LNs may not be useful for discriminating metastatic LNs from nonmetastatic LNs, particularly among relatively small LNs (e.g., <1 cm diameter).

FDG PET/CT can be used to monitor the glucose metabolism status of tissue cells, enabling the evaluation of LN metastasis according to the morphological characteristics and biological metabolism of cells. FDG PET/CT enables semiquantitative measurement of metabolism based on SUV. FDG PET/CT may be more accurate and objective than conventional CT in the diagnosis of LN metastasis [8, 32–34]; our finding was consistent with that of previous studies.

Based on logistic regression analysis, IC and SUVmax were shown to be predictors of metastatic LNs of esophageal cancer. Although the CT value can potentially reflect the extent of radiation absorption by blood vessels, the amount of enhancement indicated by the CT value may not be obvious. Relying only on the CT value might result in a less accurate evaluation of tumor metastasis to LNs. Instead, IC was determined based on a method using a three-material decomposition algorithm to generate an iodine map [35]. In the tissue, IC indicates the vascular distribution and blood supply in LNs [36], potentially distinguishing metastatic LNs from nonmetastatic LNs, similar to other tumors [36–38]. Postprocessing of dual-energy CT offers advantages in lesion assessment over conventional CT [39]. SUVmax was another independent predictor of LN metastasis in our model.

We also evaluated IC and SUVmax using the ROC curve to assess the diagnostic accuracy, further confirming the diagnostic value of IC, SUVmax, and combination of these factors for metastatic LNs. SUVmax indicates the amount of FDG metabolized by tissue. Metabolic imaging plays a role in the staging of esophageal cancer and can provide predictive and prognostic information [34]. However, SUVmax only reflects the most active part of the tumor and is not necessarily related to the entire tumor load. In the presence of other factors such as cell proliferation and tissue hypoxia, SUVmax may be affected because no SUVmax threshold has been determined to date [40, 41]. Although PET can sensitively detect tissue metabolism, its sensitivity for metastatic LNs is not high (30%–60%) [42, 43]. Although the corresponding specificity is as high as 90%, the overall evaluation is not ideal [42]. In our study, although the sensitivity and specificity of SUVmax were 88.9% and 70.3%, those of IC were 66.7% and 86.5%, respectively. Okada et al. [8] reported that ^18^F-FDG PET/CT was superior to CT because it could increase the positive predictive value to a great extent, rendering it more useful for diagnosing LN metastasis. In our study, the diagnostic performance of IC derived from dual-energy CT might be similar but slightly lower than that of SUVmax derived from FDG PET. When IC was evaluated in combination with SUVmax, the sensitivity and specificity were 83.3% and 86.5%, respectively. The AUC of the combination of IC and SUVmax was the highest, followed by the AUC of SUVmax and IC alone. Therefore, we assumed that when IC is used in combination with SUVmax, the ability to predict the presence of LN metastasis is greater than that when used individually. These parameters may have diagnostic value for metastatic LNs in patients with esophageal squamous cell carcinoma. Meanwhile, the present study results showed no correlation between IC and SUVmax in metastatic and nonmetastatic LNs. This indicates that imaging characteristics in metastatic and nonmetastatic LNs may not be constant. In other words, parameters derived from a single imaging modality may fail to characterize LNs. We assumed that combined analysis of dual-energy CT and PET/CT parameters can improve the diagnostic accuracy of LN metastasis in esophageal squamous cell carcinoma. Although the AUC of SUVmax was relatively high, diagnosis based on PET alone might be inadequate due to low spatial resolution.

This study had several limitations. First, the study sample was relatively small. Only patients who underwent surgery and were pathologically confirmed as having squamous cell esophageal carcinoma were included in this study; therefore, the number of LNs analyzed was relatively small. Second, only LNs with a short-axis diameter of ≥5 mm were analyzed, because analyzing small LNs might be unreliable with the current CT and PET imaging resolution. Thus, LN micrometastasis could not be analyzed. Third, predictive performance was only assessed for LNs that would be resected. Although the combination IC and SUVmax parameters may be a relatively better predictor than the traditional CT parameters, there is still room for improving the diagnosis of LN metastases in esophageal cancer. Further characterization of LN metastases may be required. Future studies are warranted to determine the diagnostic performance of CT and PET.

## Conclusions

Combination of dual-energy CT and PET parameters helped improve the diagnosis of LN metastasis in patients with esophageal squamous cell carcinoma.

## Supporting information

S1 Table Supplemental table shows the comparison of dual-energy CT and PET parameters between metastatic and nonmetastatic lymph nodes based on the location. (DOCX)

S1 File (XLSX)
