# Supplementary material for: Preoperative dual-energy computed tomography and positron-emission tomography evaluation of lymph node metastasis in esophageal squamous cell carcinoma
Source: PLoS One. 2024 Sep 20;19(9):e0309653. doi: 10.1371/journal.pone.0309653 (PMC11414887; doi:10.1371/journal.pone.0309653)
Supplement: S1 Table — (DOCX) [file pone.0309653.s001.docx]

**Lymph node location: neck**

| Parameters | Metastatic LNs  (n = 1) | Nonmetastatic LNs  (n = 0) | *p* |
| --- | --- | --- | --- |
| CT value (HU) | 75.2 | – | – |
| IC (mg/mL) | 1.00 | – | – |
| Fat fraction (%) | 3.55 | – | – |
| Long-axis diameter (mm) | 6.45 | – | – |
| Short-axis diameter (mm) | 5.50 | – | – |
| Ratio of long-axis to short-axis diameters | 1.17 | – | – |
| Necrosis |  |  | – |
| Present | 0 | 0 |  |
| Absent | 1 | 0 |  |
| Calcification |  |  | – |
| Present | 0 | 0 |  |
| Absent | 1 | 0 |  |
| Unclear margin |  |  | – |
| Present | 0 | 0 |  |
| Absent | 1 | 0 |  |
| Lobulated shape |  |  | – |
| Present | 0 | 0 |  |
| Absent | 1 | 0 |  |
| SUVmax | 2.3 | – | – |

**Lymph node location: supraclavicular area**

| Parameters | Metastatic LNs  (n =2) | Nonmetastatic LNs  (n =3) | *p* |
| --- | --- | --- | --- |
| CT value (HU) | 71.6 ± 4.8 | 91.3 ± 9.2 | 0.0742 |
| IC (mg/mL) | 1.40 ± 0.07 | 3.12 ± 0.43 | 0.0126 |
| Fat fraction (%) | 13.0 ± 4.6 | 23.9 ± 4.5 | 0.0791 |
| Long-axis diameter (mm) | 9.13 | 7.65 | 1.000 |
| Short-axis diameter (mm) | 4.88 | 4.45 | 0.800 |
| Ratio of long-axis to short-axis diameters | 1.85 | 1.79 | 1.000 |
| Necrosis |  |  | – |
| Present | 0 | 0 |  |
| Absent | 2 | 3 |  |
| Calcification |  |  | – |
| Present | 0 | 0 |  |
| Absent | 2 | 3 |  |
| Unclear_margin |  |  | – |
| Present | 0 | 0 |  |
| Absent | 2 | 3 |  |
| Lobulated shape |  |  | – |
| Present | 0 | 0 |  |
| Absent | 2 | 3 |  |
| SUVmax | 7.38 | 1.94 | 0.0756 |

**Lymph node location: mediastinum**

| Parameters | Metastatic LNs  (n = 4) | Nonmetastatic LNs  (n = 20) | *p* |
| --- | --- | --- | --- |
| CT value (HU) | 65.1 ± 18.5 | 84.5 ± 11.0 | 0.0086 |
| IC (mg/mL) | 1.95 ± 1.32 | 2.84 ± 0.91 | 0.1122 |
| Fat fraction (%) | 24.3 ± 10.2 | 26.7 ± 10.0 | 0.6676 |
| Long-axis diameter (mm) | 6.80 (5.58–9.78) | 8.95 (7.28–12.35) | 0.3091 |
| Short-axis diameter (mm) | 4.85 (4.48–6.30) | 5.35 (4.93–5.73) | 0.4158 |
| Ratio of long-axis to short-axis diameters | 1.40 (1.24–1.54) | 1.64 (1.33–1.95) | 0.2405 |
| Necrosis |  |  | 0.1666 |
| Present | 1 | 0 |  |
| Absent | 3 | 20 |  |
| Calcification |  |  | – |
| Present | 0 | 0 |  |
| Absent | 4 | 20 |  |
| Unclear margin |  |  | – |
| Present | 0 | 0 |  |
| Absent | 4 | 20 |  |
| Lobulated shape |  |  | 1.0000 |
| Present | 0 | 1 |  |
| Absent | 4 | 19 |  |
| SUVmax | 4.68 (3.17–5.05) | 1.79 (1.52–2.39) | 0.0134 |

**Lymph node location: hilum**

| Parameters | Metastatic LNs  (n = 0) | Nonmetastatic LNs  (n = 2) | *p* |
| --- | --- | --- | --- |
| CT value (HU) | – | 81.2 | – |
| IC (mg/mL) | – | 2.75 | – |
| Fat fraction (%) | – | 29.6 | – |
| Long-axis diameter (mm) | – | 11.5 | – |
| Short-axis diameter (mm) | – | 6.4 | – |
| Ratio of long-axis to short-axis diameters | – | 1.78 | – |
| Necrosis |  |  | – |
| Present | 0 | 0 |  |
| Absent | 0 | 2 |  |
| Calcification |  |  | – |
| Present | 0 | 0 |  |
| Absent |  | 2 |  |
| Unclear margin |  |  | – |
| Present | 0 | 0 |  |
| Absent | 0 | 2 |  |
| Lobulated shape |  |  | – |
| Present | 0 | 0 |  |
| Absent | 0 | 2 |  |
| SUVmax | – | 1.55 | – |

**Lymph node location: axilla**

| Parameters | Metastatic LNs  (n = 0) | Nonmetastatic LNs  (n = 0) | *p* |
| --- | --- | --- | --- |
| CT value (HU) | – | – | – |
| IC (mg/mL) | – | – | – |
| Fat fraction (%) | – | – | – |
| Long-axis diameter (mm) | – | – | – |
| Short-axis diameter (mm) | – | – | – |
| Ratio of long-axis to short-axis diameters | – | – | – |
| Necrosis |  |  | – |
| Present | 0 | 0 |  |
| Absent | 0 | 0 |  |
| Calcification |  |  | – |
| Present | 0 | 0 |  |
| Absent | 0 | 0 |  |
| Unclear margin |  |  | – |
| Present | 0 | 0 |  |
| Absent | 0 | 0 |  |
| Lobulated shape |  |  | – |
| Present | 0 | 0 |  |
| Absent | 0 | 0 |  |
| SUVmax | – | – | – |

**Lymph node location: intra-abdominal region**

| Parameters | Metastatic LNs  (n = 11) | Nonmetastatic LNs  (n = 12) | *p* |
| --- | --- | --- | --- |
| CT value (HU) | 76.1 ± 16.0 | 84.4 ± 17.1 | 0.2582 |
| IC (mg/mL) | 2.01 ± 0.62 | 2.76 ± 0.89 | 0.032 |
| Fat fraction (%) | 18.1 ± 7.1 | 23.5 ± 8.0 | 0.1111 |
| Long-axis diameter (mm) | 13.0 (10.9–15.1) | 8.93 (7.05–12.1) | 0.0488 |
| Short-axis diameter (mm) | 7.95 (6.58–9.61) | 4.95 (4.75–6.76) | 0.0526 |
| Ratio of long-axis to short-axis diameters | 1.73 (1.36–2.02) | 1.71 (1.30–1.99) | 0.9279 |
| Necrosis |  |  | 0.5900 |
| Present | 2 | 1 |  |
| Absent | 9 | 11 |  |
| Calcification |  |  | – |
| Present | 11 | 0 |  |
| Absent | 0 | 12 |  |
| Unclear margin |  |  | 0.3167 |
| Present | 3 | 1 |  |
| Absent | 8 | 11 |  |
| Lobulated shape |  |  | – |
| Present | 3 | 1 | 0.3167 |
| Absent | 8 | 11 |  |
| SUVmax | 2.80 (2.14–3.43) | 1.48 (1.20–1.88) | 0.0336 |
